# Two-Year Outcomes for Patients with Non-ST-Elevation Acute Coronary Syndrome Treated with Magmaris and Absorb Bioresorbable Scaffolds in Large-Vessel Lesions

**DOI:** 10.3390/jpm14050540

**Published:** 2024-05-17

**Authors:** Adrian Włodarczak, Piotr Rola, Szymon Włodarczak, Marek Szudrowicz, Katarzyna Giniewicz, Magdalena Łanocha, Joanna Jaroszewska-Pozorska, Mateusz Barycki, Łukasz Furtan, Michalina Kędzierska, Piotr Włodarczak, Adrian Doroszko, Maciej Lesiak

**Affiliations:** 1 Department of Cardiology, The Copper Health Centre (MCZ), 59-300 Lubin, Poland; wlodarczak.adrian@gmail.com (A.W.); wlodarczak.szy@gmail.com (S.W.); marek.szudrowicz@gmail.com (M.S.); asiulaj@gmail.com (J.J.-P.); piotrwlodarczak123@gmail.com (P.W.); 2 Department of Cardiology, Provincial Specialized Hospital in Legnica, 59-220 Legnica, Poland; mateusz.barycki@gmail.com (M.B.); lukas.furtan@gmail.com (Ł.F.); 3 Faculty of Health Sciences and Physical Culture, Witelon Collegium State University, 59-220 Legnica, Poland; 4 Independent Researcher, 50-556 Wroclaw, Poland; katarzyna@giniewicz.it; 5 Adalbert’s Hospital, 61-144 Poznan, Poland; mlanocha@hotmail.com; 6 Faculty of Medicine, Wroclaw Medical University, 50-556 Wroclaw, Poland; kedzierska.michalina@gmail.com; 7 Department of Cardiology, Center for Heart Diseases, 4th Military Hospital, Faculty of Medicine, Wroclaw University of Science and Technology, 50-981 Wroclaw, Poland; adrian.doroszko@gmail.com; 8 1st Department of Cardiology, Poznan University of Medical Sciences, 61-491 Poznan, Poland; maciej.lesiak@skpp.edu.pl

**Keywords:** Magmaris, Absorb, bioresorbable scaffolds, acute coronary syndrome, magnesium scaffolds, percutaneous coronary intervention, mid-term

## Abstract

Background: The acute coronary syndrome (ACS) continues to be a fundamental indication for revascularization by percutaneous coronary intervention (PCI). Drug-eluting stent (DES) implantation remains a part of contemporary practice but permanent caging of the vascular structure with the metallic stent structure may increase the rate of device-related adverse clinical events. As an alternative to classic metallic DESs, the bioresorbable scaffolds (BRSs) have emerged as a temporary vascular support technology. We evaluated the mid-term outcomes of two generations of bioresorbable scaffolds—Absorb (Abbott-Vascular, Chicago, IL, USA) and Magmaris (Biotronik, Germany)—in patients with non-ST-elevation ACS. Methods: The study cohort consisted of 193 subjects after Magmaris implantation and 160 patients following Absorb implantation in large-vessel lesions. Results: At 2 years, a significantly lower rate of a primary outcome (cardiac death, myocardial infarction, stent thrombosis) was observed with Magmaris (5.2% vs. 15%; *p* = 0.002). In addition, we observed a significantly lower rate of MI in the target vessel (2.6% vs. 9.4%; *p* = 0.009) and a lower rate of scaffold thrombosis (0% vs. 3.7%; *p* = 0.008). The TLF rate between the two groups was not significantly different. Conclusion: Magmaris demonstrated a good safety profile and more favorable clinical outcomes when compared to Absorb in patients with non-ST-elevation ACS.

## 1. Introduction

Acute coronary syndrome (ACS) remains a fundamental indication for percutaneous coronary intervention (PCI). Despite undeniable improvements in the design, construction, and biocompatibility of the DES technology, the permanent caging of the vascular structure with the metal structure is still associated with an increased rate of device-related adverse clinical events [1]. The DES is implanted during ACS-PCI to maintain the patency of the artery during the healing process. After an acute period, the DES becomes redundant and may lead to several adverse events such as restenosis, thrombosis, or late lumen loss. The origin of this process seems to be related to a prolonged enhanced local inflammatory process [2]. Theoretically, the bioresorbable scaffolds (BRSs) have emerged as an alternative to DESs. The basic assumptions of this medical concept focus on providing initial vascular integrity with a delayed complete resorption of a scaffold and subsequent dismission of the prolonged local inflammatory process [3]. However, long-term data of the first generation of BRS–Absorb (Abbott-Vascular, Chicago, IL, USA) suggest less favorable results compared to leading DESs [4,5,6]. The background of this observation is multifactorial and partly unclear [7,8]. 

Despite the initial drawbacks, the BRS concept still evokes attention. Recently, a novel magnesium bioresorbable scaffold—Magmaris (Biotronik, Berlin, Germany)—has been introduced to clinical practice and showed favorable clinical results in terms of short-term observation [9,10,11,12,13,14]. However, to understand the differences between the two generations of BRSs and to objectively assess the clinical significance of these differences, it is essential to compare the efficacy of Magmaris with its predecessor, the Absorb scaffold. In this retrospective observational study, we evaluated the 2-year safety and efficacy of two generations of BRSs (Magmaris vs. Absorb) in patients with non-ST-elevation ACS, treated in large-vessel lesions with an optimal implantation technique [15].

This study collected the data from all consecutive patients who underwent BRS implantation at our cardiac center (Copper Heath Center, Lubin, Poland) and met the study inclusion and exclusion criteria. The first group consisted of 160 patients who received at least one Absorb (Abbott Vascular, Chicago, IL, USA) between April 2012 and August 2017, while the second group consisted of 193 patients who received one or more Magmaris (Biotronik, Germany, Berlin) between October 2016 and March 2020.

## 2. Materials and Methods

### 2.1. Study Population and PCI Procedures

**Figure 1:**
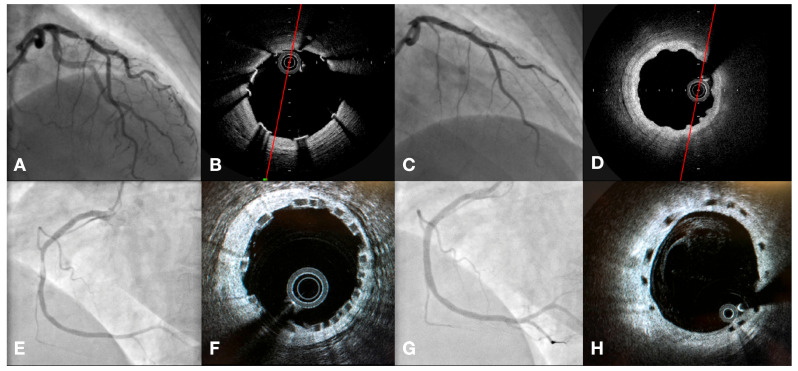
Exemplary Magmaris and Absorb BRS implantation procedure. (**A**)—initial angiogram. (**B**)—OCT result after Magmaris implantation. (**C**)—final angiogram after Magmaris implantation. (**D**)—OCT assessment 12 months after Magmaris implantation. (**E**)—initial angiogram of second lesion. (**F**)—OCT result after Absorb implantation. (**G**)—final angiogram after Absorb implantation. (**H**)—OCT assessment 12 months after Absorb implantation.

This investigator-initiated, single-center, double-arm, observational study includes pooled data from patients undergoing percutaneous coronary intervention with lesions suitable for BRSs who received a first- or second-generation BRS. All implantations were performed between April 2012 and March 2020. This investigator-initiated, single-center, double-arm, observational study includes pooled data from patients undergoing percutaneous coronary intervention with lesions suitable for BRSs who received a first- or second-generation BRS. This study included patients admitted to our Heart Center with an initial diagnosis of ACS, excluding cases of ST-elevation acute myocardial infarction (STEMI). The initial diagnosis was based on clinical assessment by the trained medical staff in combination with additional investigations (ECG; cardiac marker assessment). The diagnosis of non-ST-elevation acute myocardial infarction (NSTEMI) was made according to the third or fourth universal definition of infarction, depending on the time of initial treatment. Despite the initial diagnosis of ACS, subjects enrolled in this study met several additional criteria—the target lesion was suitable for magnesium BRS implantation (vessel reference diameter in the range of 2.7 mm to 3.7 mm and lesion length less than 21 mm), and the main exclusion criteria were the clinical presentation of STEMI with a high thrombus burden and presence of TIMI 0 flow at the beginning of the procedure. The complete list of study inclusion and exclusion criteria along with a full discussion of all aspects of the study design has been published previously [16,17]. Briefly, this study consisted of two ACS cohorts; the first group included 193 patients treated by the implantation of the Magmaris BRS. The second group comprised 160 patients who received at least one Absorb BRS during the PCI procedure. For this study, we recruited patients with large-vessel diseases (diameter: 3.0 mm or higher) whose BRS implantation procedure was followed by the “4P” strategy. Basic principles of the “4P” strategy are key to [18], proper sizing, adequate lesion selection, and preparation (mandatory initial, aggressive pre-dilatation with a non-compliant (NC) balloon catheter sized equally to treated vessel diameter (1:1 balloon//artery ratio), and followed by mandatory post-dilatation with non-compliant ballon catheter (prolonged, high-pressure inflation with at least 16 atm with a balloon sized at least with 1:1 balloon/scaffold ratio or up to 0.5 mm longer). The “4P” strategy was applied in both study arms (Magmaris and Absorb). Exemplary Magmaris and Absorb BRS implantation procedures are presented in Figure 1.

### 2.2. Study Devices

Magmaris, previously known as DREAMS 2G, is the bioresorbable magnesium scaffold CE marked in Europe since June 2016. It is a bioresorbable metal scaffold that is coated with a BIOlute poly-L-lactide (PLLA) biodegradable polymer that elutes sirolimus. PLLA is biocompatible and capable of self-catalyzing hydrolytic degradation to lactic acid. The drug release time is calibrated for approximately 90 days, whereas the PLLA resorption time is 2 years. The Magmaris device’s backbone is completely radiolucent. To navigate the scaffold implantation under an X-ray, two permanent tantalum radiopaque markers are attached to the distal and proximal ends. The average complete scaffold resorption time is approximately one year. Magmaris has an average strut thickness of 150 μm and is available in diameters of 3.0 and 3.5 mm and lengths of 15, 20, and 25 mm. 

The ABSORB BVS (Abbott-Vascular, Chicago, IL, USA) backbone was constructed of poly-L-lactic acid covered with an everolimus-eluting polymer, both of which resorb in approximately 3 years. The average strut thickness is 150 μm. The device is available in a wide range of diameters and lengths (diameters from 2.5 to 3.5 mm and lengths from 8 to 28 mm), but in this study, we only included scaffolds within the size corresponding to Magmaris (diameters 3.0 mm or 3.5 mm and lengths 12, 18, or 24 mm).

### 2.3. Study Endpoint and Follow-Up

The primary outcome was composed out of cardiovascular death, myocardial infarction, and definite or probable in-stent thrombosis. The primary secondary outcome was target lesion failure (TLF), defined as cardiac death, target vessel myocardial infarction (TV-MI), or target lesion revascularization (TLR). The time points for the evaluation were 1 year and 2 years after the index procedure. Telephone contact and/or personal scheduled visits to the cardiac center were used to evaluate patients during the follow-up. 

Clinical component endpoints were based on Academic Research Consortium definitions [19] and included death, MI, target lesion revascularization (TLR), target vessel revascularization (TVR), total coronary revascularization, and scaffold thrombosis and restenosis. Myocardial infarcts are defined by the Fourth Universal Definition of Myocardial Infarct [20]. 

### 2.4. Statistical Analysis

Means and standard deviations are reported for continuous variables and frequency for categorical variables. The nonparametric two-sample Mann–Whitney test for continuous variables and Fisher’s exact test for categorical variables were used to compare study cohorts. To adjust for multiple comparisons, the Bonferroni correction was applied. For variables that reached statistical significance in the univariate analysis, a multivariate Cox analysis was performed. Statistical significance was defined as *p*-values ≤ 0.05. All statistical analyses were performed using the R language by a professional statistician, similar to medical analyses.

## 3. Results

The baseline clinical and procedural characteristics of both study cohorts have been well described [10,11,12,13,14,16,17,21]; however, we present all data in Table 1 and Table 2. In brief, this study consisted of two arms. A Magmaris BRS was implanted in 193 patients and 160 patients treated with an Absorb BRS were enrolled. In the Magmaris group, we observed a statistically higher prevalence of NSTEMI compared to the Absorb group (84.5% vs. 60.6%; *p* < 0.001). Additionally, this cohort had a significantly lower rate of LAD target vessel PCI (41.4% vs. 52.1%; *p* = 0.036). There were no significant differences in comorbidities between the two groups. The only difference in laboratory parameters between the study groups was observed in the serum lipid and creatine levels.

A significantly lower prevalence (56.9% vs. 62.6%; *p* < 0.001) of a 3.5 mm scaffold size in the Magmaris arm was observed. On the one hand, we observed a significantly higher diameter of the balloon used for post-dilatation in the Magmaris arm, and on the other hand, we observed a significantly lower pressure used during post-dilatation (mean pressure (atm): 17.7 ± 0.8 vs. 18.2 ± 2.5; *p* < 0.001), which is likely reflected in a significantly lower rate of vessel perforation during PCI (0 vs. 4; *p* = 0.041). Additionally, ticagrelor was more often used as a part of dual antiplatelet therapy in the Magmaris cohort when compared to Absorb (60.6% vs. 21.8%; *p* < 0.001)

In addition, the univariable Cox regression analysis was performed to evaluate potential factors that could have an impact on the primary outcomes. Subsequently, characteristics that reached statistical significance (*p* < 0.05) were included in the multivariable Cox regression model. None of them showed a significant impact on the observed outcome. 

As it was previously reported at the 1-year follow-up, we observed a significantly lower rate of the primary outcome in the Magmaris group (1.5% vs. 8.1%; *p* = 0.003) along with a lower number of TLFs (1.5% vs. 5.6%; *p* = 0.042). In addition, we observed a lower number regarding target vessel MI (1.0% vs. 5.6%; *p* = 0.026) and scaffold thrombosis (0% vs. 3.7%; *p* = 0.008). 

**Figure 2:**
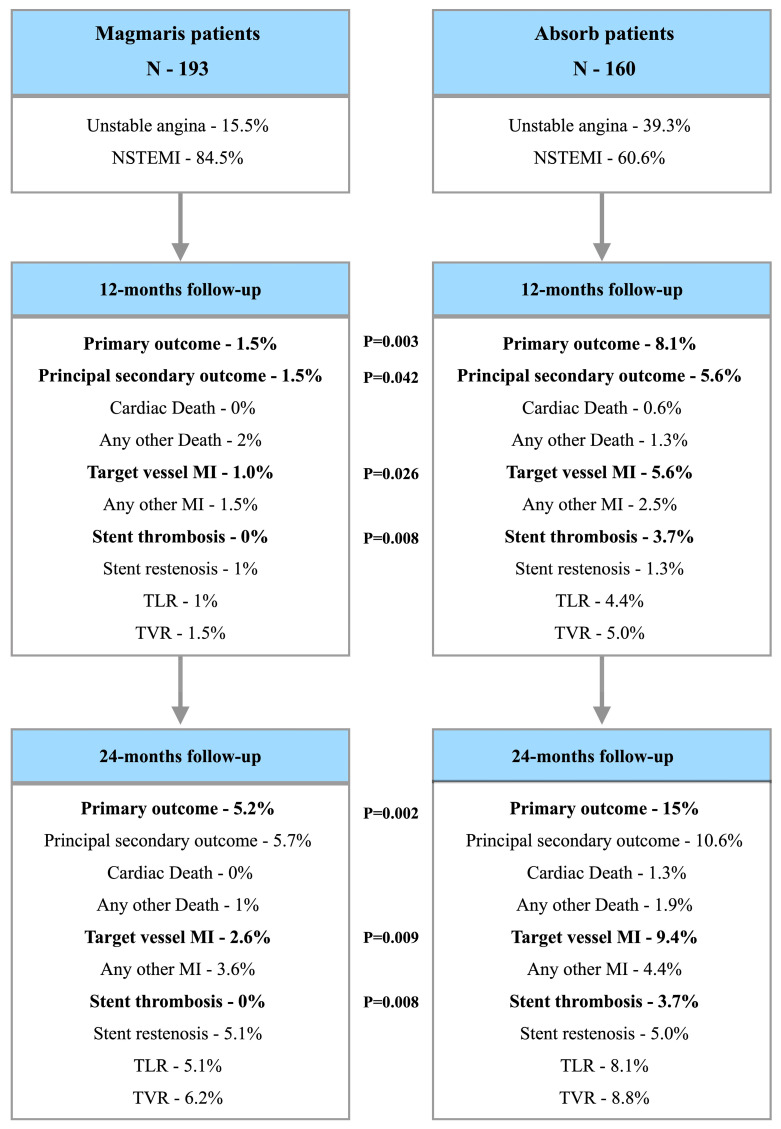
Study flow-chart.

In terms of the 2-year follow-up, the significantly lower rate of the primary outcome was still observed in the Magmaris arm (5.1% vs. 15.0%; *p* = 0.002); however, in contrast to the 1-year follow-up, we did not observe a significant difference in the TLF rate between both study groups. Similar to the 1-year observation, we observed a significantly lower number regarding target vessel MI (2.6% vs. 9.4%; *p* = 0.009) and scaffold thrombosis (0% vs. 3.7%; *p* = 0.008). All clinical outcome data are summarized in Table 3; additionally, a study flow-chart is presented in Figure 2.

**Figure 3:**
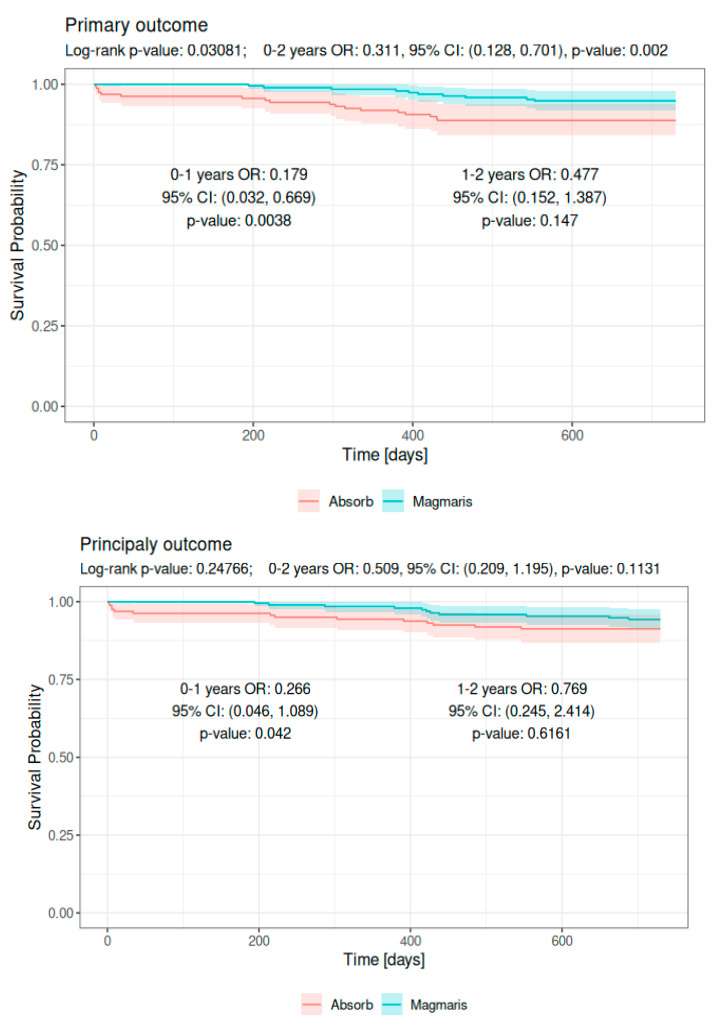
Kaplan–Meier curves regarding primary and principal secondary outcome.

In addition, we performed an additional landmark analysis (referring to the period between 1 and 2 years after implantation) on two key study endpoints, which are presented in Table 4. Figure 3 shows Kaplan–Meier curves for the primary and secondary endpoints.

## 4. Discussion

The key findings of our study are as follows: (1) Magmaris compared to Absorb in the NSTE-ACS cohort showed a significantly lower rate of the primary endpoint (death from cardiac causes, myocardial infarction, stent thrombosis) in pooled data at 2 years of the follow-up; however, the landmark analysis at 1–2 years of this study showed no significant differences between the two study groups.

(2) The implantation of Magmaris compared to Absorb was associated with a significantly lower rate of stent thrombosis and target-vessel-related MI at the 2-year follow-up; however, no significant differences were noted in terms of TLR.

(3) No definite scaffold thrombosis was reported in the Magmaris cohort at the 2-year follow-up. 

Since their introduction into clinical practice, second-generation drug-eluting stents (DESs) have become the gold standard in percutaneous coronary intervention (PCI). Despite the undeniable improvement in the clinical outcome of modern DESs compared to BMS or the first generation of DESs [22], there are still some shortcomings associated with the permanent caging of the vessel with a metal backbone [23]. To overcome these limitations and potentially reduce long-term adverse events, the concept of bioresorbable scaffolds has been developed. The first generation of bioresorbable scaffolds has been represented by the Absorb BRS and has been widely introduced to clinical practice. Despite initial enthusiastic results, long-term observation revealed several shortcomings of this technology, mainly associated with increased rates of stent thrombosis, which ultimately led to the withdrawal of this scaffold from the market. 

Several potential factors have been postulated to be involved in the etiopathogenesis of device failure. Absorb devices tend to have greater strut thickness, high thrombogenicity, and lower radial force, which, combined with delayed endothelialization time and prolonged, unpredictable bioabsorption, result in scaffold backbone degradation, leading to the disintegration of the stent architecture associated with late strut protrusion into the vessel lumen. Bench testing and initial clinical evaluation [24,25,26] suggest that the second-generation BRS—a magnesium scaffold (Magmaris)—has succeeded in overcoming the aforementioned limitation of polymeric scaffolds. Specifically, Magmaris was shown to be up to 80% more flexible, require up to 40% less lesion entry force, and have better pushability compared to the Absorb polymer scaffold [24]. These data, combined with the low thrombogenicity of magnesium ions and the good radial strength of Magmaris [3], suggest a potential clinical advantage of the magnesium BRS over its predecessor.

However, there is a paucity of data directly comparing the two generations of BRSs. Previously published 1-year data from our registry suggested a significantly lower rate of the primary outcome in the Magmaris group (1.5% vs. 8.1%; *p* = 0.003) along with a lower number regarding TLF (1.5% vs. 5.6%; *p* = 0.042) and scaffold thrombosis (0% vs. 3.7%; *p* = 0.008) [16]. Nevertheless, a significant difference in the primary outcome and scaffold thrombosis was maintained; still, there was a noticeable trend in terms of TLF (5.7% vs. 10.6%) in favor of Magmaris, but it did not reach statistical significance at the 2-year follow-up (*p* = 0.112).

It is important to emphasize that in both study cohorts, the “4P strategy” was used (proper patient and lesion selection; aggressive pre-dilation and post-dilation; precise sizing (large vessel diameter of at least 3 mm)), which has been shown to have a strong positive impact on long-term outcomes after BRS implantation. This restrictive approach to scaffold implantation may overcome the limitations of the device and is probably one of the key issues that will determine the fate of BRS technology [27,28,29].

Several large observational registries as well as a few mid-sized studies suggest good clinical outcomes similar to our study subpopulation with TLF not exceeding 7% at 2 years of the follow-up [30,31,32,33,34]. Furthermore, scaffold thrombosis is a marginal phenomenon in all mentioned studies. If we confront these data with the pooled data from the Absorb trials, where TLF reached 9.3% with a concomitant stent thrombosis rate of 2.3% [35], we could assume that each BRS is different and that there is no class effect.

### Limitations

Our study has several limitations, including the retrospective observational nature of this study with all the inherent drawbacks of such studies. The second limitation is the single-center recruitment model with a relatively small number of participants. We observed significant differences in baseline clinical characteristics of both study groups (without significant impact on primary outcome in the multivariable Cox regression model). Moreover, despite the high prevalence of clopidogrel postprocedural pharmacological therapy in both study cohorts, we did not perform a test to evaluate potential resistance to this drug.

## 5. Conclusions

Mid-term data (2-year follow-up) from our study suggest fewer adverse events after Magmaris implantation compared to Absorb in the ACS cohort. No definite scaffold thrombosis occurred in the ACS–BRS–Magmaris population during the observation period.

## Figures and Tables

**Table 1:** Study group baseline clinical characteristics.

	Magmaris N—193	Absorb N—160	*p*-Value
Age	66.3 ± 8.9	65.8 ± 9.7	*p* = 0.244
Gender—male (ratio)	150 (77.7%)	117 (73.1%)	*p* = 0.32
Unstable angina	30 (15.5%)	63 (39.3%)	*p* < 0.001
NSTEMI	163 (84.5%)	97 (60.6%)	*p* < 0.001
Diabetes	72 (37.3%)	61 (38.1%)	*p* = 0.912
Oral anti-diabetic drug	58 (30%)	48 (30%)	*p* = 1
Insulin use	14 (7.2%)	13 (8.1%)	*p* = 0.841
Hypertension	171 (88.6%)	131 (81.8%)	*p* = 0.094
Hypercholesterolemia	152 (78.7%)	133 (83.1%)	*p* = 0.343
Atrial fibrillation	9 (4.6%)	5 (3.1%)	*p* = 0.587
Post-PCI status	78 (40.4%)	58 (36.2%)	*p* = 0.443
Past MI	59 (30.5%)	50 (31.2%)	*p* = 0.908
Tobacco smoker	57 (29.5%)	52 (32.5)	*p* = 0.565
LVEF	60.4% ± 10.9	55.6% ± 13.2	*p* < 0.001
Total cholesterol (mmol/L)	4.6 ± 1.3	5.1 ± 1.3	*p* = 0.006
LDL (mmol/L)	2.5 ± 1.2	2.9 ± 1.2	*p* = 0.004
Triglycerides (mmol/L)	1.8 ± 1.8	2.0 ± 1.4	*p* = 0.232
Creatinine (µmol/L)	84.1 ± 22.2	87.7 ± 17.2	*p* = 0.010
Duration of hospitalization (days)	2.7 ± 1.8	3.4 ± 2.7	*p* = 0.013

Abbreviations: NSTEMI, non-ST-elevation myocardial infarction; PCI, percutaneous coronary intervention; MI, myocardial infarction; LVEF, left ventricle ejection fraction.

**Table 2:** PCI procedure features in both cohorts.

Procedural Characteristic	Magmaris N—193	Absorb N—160	*p*-Value
Treated vessel: LAD	80 (41.4%)	88 (52.1%)	*p* = 0.036
LCx	49 (25.3%)	24 (14.2%)	*p* = 0.036
RCA	61 (31.6%)	57 (33.7%)	*p* = 0.430
IM	3 (1.6%)	0 (0%)	*p* = 0.339
Pre-dilation balloon:			
Mean diameter (mm)	3.2 ± 0.3	3.1 ± 0.3	*p* = 0.092
Mean pressure (atm)	17.7 ± 0.8	16.8 ± 1.9	*p* = 0.067
Average scaffold number	1.1 ± 0.2	1.3 ± 0.5	*p* = 0.343
Scaffold diameter: 3.0 (mm)	88 (43.1%)	76 (37.4%)	*p* = 0.748
3.5 (mm)	116 (56.9%)	127 (62.6%)	*p* < 0.001
Average scaffold length (mm)	20.8 ± 3.3	22.7 ± 4.8	*p* = 0.002
Post-dilation balloon:			
-Mean diameter (mm)	3.5 ± 0.3	3.5 ± 0.3	*p* = 0.067
-Mean pressure (atm)	17.7 ± 0.8	18.2 ± 2.5	*p* < 0.001
-0.0 mm greater than scaffold	31 (16.1%)	70 (43.8%)	*p* < 0.001
-0.25 mm greater than scaffold	130 (67.3%)	64 (40%)	*p* < 0.001
-0.5 mm greater than scaffold	32 (16.6%)	26 (16.2%)	*p* = 0.998
Syntax score	7.7 ± 4.2	7.9 ± 4.5	*p* = 0.718
Amount of contrast used (mL)	151.5 ± 65.4	169.1 ± 58.0	*p* < 0.001
Radiation dose (mGy)	1056.7 ± 697.8	1551.0 ± 853.3	*p* < 0.001
IVUS/OCT-guided PCI	41 (21.2%)	21 (13.1%)	*p* = 0.052
Recognized edge dissection:	7 (3.6%)	8 (5%)	*p* = 0.601
-Treated with additional BRS (Magmaris/Absorb)	3 (1.5%)	6 (3.7%)	*p* = 0.310
-Treated with DES	4 (2.0%)	2 (1.2%)	*p* = 0.693
Vessel perforation—covered stent implantation	0 (0%)	4 (2.5%)	*p* = 0.041
-Prolong balloon inflation	0 (0%)	3 (1.9%)	*p* = 0.092
	0 (0%)	1 (0.6%)	*p* = 0.453
Occlusion of the side branch	2 (1%)	1 (0.6%)	*p* = 0.989
Antiplatelet therapy: ASA	193 (100%)	160 (100%)	-
Clopidogrel	76 (39.4%)	122 (76.3%)	*p* < 0.001
Ticagrelor	117 (60.6%)	35 (21.8%)	*p* < 0.001
Prasugrel	0 (0%)	3 (1.9%)	*p* = 0.092

Abbreviations: OCT, optical coherence tomography; PCI, percutaneous coronary intervention; ASA, acetylsalicylic acid; DES, drug-eluting stent; BRS, bioresorbable vascular scaffold.

**Table 3:** Clinical results in both cohorts.

Clinical Outcomes	Magmaris N—193	Absorb N—160	*p*-Value
1-Year Follow-Up			
Primary outcome: (myocardial infarction, cardiac death, stent thrombosis)	3 (1.5%)	13 (8.1%)	*p* = 0.003
Principal secondary outcome:	3 (1.5%)	9 (5.6%)	*p* = 0.042
Target lesion failure (target vessel myocardial infarction, cardiac death, target lesion revascularization)			
Death:			
-Any other	2 (1.0%)	2 (1.3%)	*p* = 1
-Cardiac	0 (0%)	1 (0.6%)	*p* = 0.453
Myocardial infarction:			
-Any other	3 (1.5%)	4 (2.5%)	*p* = 0.706
-Target vessel	2 (1.0%)	9 (5.6%)	*p* = 0.026
Scaffold:			
-Thrombosis	0 (0%)	6 (3.7%)	*p* = 0.008
-Restenosis	2 (1.0%)	2 (1.3%)	*p* = 1
Stroke	2 (1.0%)	4 (2.5%)	*p* = 0.416
TIA	1 (0.5%)	0 (0%)	*p* = 1
Revascularization:			
-Target lesion	2 (1.0%)	7 (4.4%)	*p* = 0.084
-Target vessel	3 (1.5%)	8 (5.0%)	*p* = 0.072
-Any other	18 (9.3%)	16 (10.0%)	*p* = 0.857
2-Year Follow-Up			
Primary outcome: (myocardial infarction, cardiac death, stent thrombosis)	10 (5.2%)	24 (15%)	*p* = 0.002
Principal secondary outcome:	11 (5.7%)	17 (10.6%)	*p* = 0.112
Target lesion failure (target vessel myocardial infarction, cardiac death, target lesion revascularization)			
Death			
-Any other	2 (1.0%)	3 (1.9%)	*p* = 0.660
-Cardiac	0 (0%)	2 (1.3%)	*p* = 0.201
Myocardial infarction:			
-Any other	7 (3.6%)	7 (4.4%)	*p* = 0.786
-Target vessel	5 (2.6%)	15 (9.4%)	*p* = 0.009
Scaffold:			
-Thrombosis	0 (0%)	6 (3.7%)	*p* = 0.008
-Restenosis	10 (5.1%)	8 (5.0%)	*p* = 1
Stroke	2 (1%)	5 (3.1%)	*p* = 0.250
TIA	1 (0.5%)	0 (0%)	*p* = 1
Revascularization:			
-Target lesion	10 (5.1%)	13 (8.1%)	*p* = 0.283
-Target vessel	12 (6.2%)	14 (8.8%)	*p* = 0.414
-Any other	23 (11.9%)	19 (11.9%)	*p* = 1

Abbreviations: TIA, transient ischemic attack; MI, myocardial infarction.

**Table 4:** Results of the Landmark analysis regarding period between 1 and 2 years of this study.

Clinical outcomes	Magmaris Patients N—189	Absorb Patients N—147	*p*-Value	OR	95% CI
1–2-Year FU Primary Outcome: (cardiac death, myocardial infarction, stent thrombosis)	7 (3.7%)	11 (7.5%)	*p* = 0.147	0.476	0.476 (0.152–1.387)
1–2-Year FU Principal Secondary Outcome: Target lesion failure (cardiac death, target vessel myocardial infarction, target lesion revascularization)	8 (4.2%)	8 (5.4%)	*p* = 0.616	0.768	0.767 (0.244–2.413)

Abbreviations: OR, odds ratio; CI, confidence interval.

## Data Availability

Data not included in manuscript available on request from corresponding author due to local law and privacy restrictions.
